# The Institutional Worker

**Published:** 1913-08-16

**Authors:** 


### INSTITUTIONAL WORKER

**Being a Special Supplement to "The Hospital"**

### OUR BUREAU OF INFORMATION.

### Rules for Correspondents.

1. Every letter must be accompanied by the coupon to be cut from the back cover (inside page) of The Hospital, current issue, and must contain the name and address of the correspondent with pseudonym for publication if desired. Replies by post cannot be given, save under exceptional circumstances at the Editor's discretion.

2. Letters from Approved Homes in reply to special needs published in the Bureau should state terms and full particulars, and be sent prepaid under cover to the Editor of the Bureau with name written across coupon for identification.

3. Proprietors of Homes which have not yet been entered on the List of Approved Homes, but have spare accommodation likely to suit special needs, are invited to write for an application form for registration. The fee for registration, which includes two announcements of the Home in the Bureau and other privileges, is 10s.

4. All communications to be addressed to the Editor of The Hospital, 28 Southampton Street, Strand, London, W.C., and marked "Bureau of Information."

### INSTITUTIONAL FACTS AND FIGURES.

Several interesting and instructive replies have been received to Question 2 for July, which was: If you keep your accounts in accordance with the Uniform System, describe fully your method of checking, sorting, analysing, and filing your accounts for payment. Give such particulars as will clearly indicate the manner in which these are passed through your analysis journal, and onwards through the ledger. Add your further remarks. The replies indicate that the importance of this work is very fully appreciated by the responsible officers of the institutions in question. Methods of procedure necessarily vary in accordance with the character and size of the institution, but, after taking into consideration these circumstances, a comparison of the methods should be helpful to hospital officers in elucidating their difficulties and in evolving the most efficient system of dealing with the work. Space permits us to publish only one answer this week, but we hope to publish others in later issues.

### ACCOUNT CHECKING AT A LONDON HOSPITAL.

#### First Selected Answer.

All invoices without exception go to the assistant secretary, who classifies them every morning under the departments, or the officers concerned with the goods mentioned in them—as matron, resident medical officer, dispensary, etc. They are then stamped with a rubber stamp in this form:—

| | Initials |
|---|---|
| Goods Received .............. | |
| Prices Correct .............. | |
| Invoice Checked and Compared with Order .......... | |

The invoices are then entered roughly in a book with columns ruled and headed to correspond with the departments or persons to whom the batches of invoices will be sent, and this book is sent round with the bills. The duty of each recipient is, of course, to initial for such of the points as his responsibility or knowledge covers. Thus the matron may initial for the receipt of bedding and linen, and yet have no knowledge of the prices to be charged; on the other hand, surgical instruments may be sent straight to the theatre, so that the resident medical officer would not know whether they had been received, though able to vouch for the prices. In cases where the officer concerned is able to deal with the invoices at once, he does so, crosses them off the invoice book, and returns them to the bearer. If, on the other hand, he has to keep them for some little time, he merely initials the book for their receipt. If at the end of the month any invoice is found to be missing, the book thus provides a ready means of tracing it. When the invoices, duly initialled, have been received back into the secretary's office, the points covered by the third line of the rubber stamp are dealt with. In the course of this, the counterfoil of the order book is crossed through, or otherwise marked, to prevent two charges being put through for the same goods. At the end of the month the counterfoils are examined to see that all the invoices have been received, as the late arrival or accidental loss of an invoice may seriously prejudice the comparison of one month's expenditure with another.

##### The Bought Day Book.

When the assistant secretary or book-keeper is satisfied that all the bills have been received, he sorts them into order of date (or into alphabetical order if more convenient), and enters them into the bought day book, in the course of which process each invoice receives a number. The invoices thus numbered are placed in a dust-proof but easily accessible file, on the back of which is marked plainly the month and the numbers included therein. The bought day book has columns only for the main heads of expenditure, and it is therefore necessary to dissect the charges into the sub-heads.

##### Entering the Analysis Book.

With the analysis book opened at (for example) "Provisions," and the file of invoices at his side, the book-keeper runs down the provisions column of the bought day book, and does the dissection quickly and easily, while at the end of the process the invoices remain filed as at the beginning. The two books are, of course, cross checked by the addition.

##### Posting the Ledger.

From the bought day book the charges are entered into the personal ledger, and against each entry is given not only the folio of the day book but the number of the invoice, this being a very useful time-saver when references have to be made. The personal ledger is balanced off quarterly, after the statements rendered by the tradesmen have been checked. At the same time the totals under the main heads are posted to separate accounts in the impersonal ledger, this containing *per contra* the receipts from various sources which go to the credit of the different heads. Thus payments by patients for bottles will be placed to the credit of the surgery and dispensary account, and when the total of such payments has been deducted from the amount posted from the bought day book, the balance is the amount to be carried to the income and expenditure account.

##### Advantage of Personal Ledger.

The personal ledger is often omitted, but this book is not only necessary by the theory of accountancy, but it is of value and assistance in many directions. For instance, it enables one to answer within two minutes such a question as this: "We bought a sterilising drum some time within the last three years off Jones and Co., with whom we have not had any other dealings. How much did we pay for it?"

##### General Remarks.

The system thus outlined is more complete than is the case in many hospitals, but there is no link in the chain which could be omitted without the risk of loss of efficiency. The bought day book and the analysis book might, and in some cases have been, thrown into a single volume, but there are several practical disadvantages to be urged against this procedure. With regard to invoices, it is important that the points specified in the rubber stamp given above should be dealt with separately, and a signature obtained under each heading.—"Shamus."

### QUESTIONS for AUGUST.

AUGUST is a holiday-making time, and therefore the questions we are publishing for this month are of such a nature as to be easy to answer without recourse to reference books. Question 2 touches on a branch of philanthropy which is coming greatly to the fore, and practical workers in this field are invited to give their experience which cannot fail to be of considerable help to the many beginners whom this work is attracting. Question 1 should appeal to the secretariat of any institution, and their views, with the reasons for such views, will be welcome.

**Any Use for an Addressograph ?**

**1. Is it or is it not an advantage for a hospital to possess an addressograph? Give reasons, and if an advantage give particulars of cost.**

**Mothers' Dinners Menus.**

**2. Give a week's menu for dinners for mothers in connection with Schools for Mothers, with the daily cost for 20 people, bearing in mind the need for nourishing food and for strict economy.**

RULES.

The following rules must be observed:—

1. Contributions must be written on one side of the paper. They must bear the name and address of the sender and be accompanied by coupon to be cut from the back cover (inside page) of the current issue of THE HOSPITAL. A pseudonym must be chosen if the name is not to be published.

2. They must be addressed to the Editor of THE HOSPITAL, 28 & 29 Southampton Street, Strand, London, W.C., so as to reach him by the last day of the month for which the questions are set, and must be marked in the left-hand corner "Facts and Figures."

**A minimum payment of Five Shillings will be made for each published answer.**

### *THE SICK AND IN NEED.*

**Approved Home.**

WE have pleasure in adding the following Home to our Approved List:—

NORFOLK.—The Bungalow, Long Stratton. Principal: Nurse Marion W. Pike. A suitable home for a delicate or deficient child or a maternity case. Special advantages: Massage, rest-cure, and open-air treatment. Inclusive terms for a child, 30s. per week; for a maternity case, £5 per week. A doctor visits daily if necessary.

**Phthisical Child.**

THERE are very few sanatoria which receive cases free of charge. Apply to the Lady Superintendent, Salop Convalescent Home for Women and Children, at Baschurch, Shrewsbury; phthisical girls between three and fifteen years of age are treated here free upon the production of a subscriber's letter, or for 8s. 6d. per week. Girls from two years of age are also received at the Children's Sanatorium, Holt, Norfolk, for a weekly payment of 7s. 6d.; application should be made to the Hon. Sec., 68 Denison House, Vauxhall Bridge Road, London, S.W.—C. T. B.

### THE EDITOR'S LETTER-BOX.

### *EMPLOYMENT AND TRAINING.*

**Irish Appointment.**

ANNOUNCEMENTS of vacancies for nurses at Irish institutions appear in the advertisement columns of *The Nursing Mirror* and other nursing journals from time to time; you should watch for these and apply for them. A full list of Irish hospitals is given in "Burdett's Hospitals and Charities," published by The Scientific Press, Ltd., 28 and 29 Southampton Street, Strand, London, W.C.—"NURSE H."

**Bermondsey Medical Mission.**

YOU will find many opportunities in London of gratifying your desire to take part in social work amongst the sick poor, but this work cannot be properly approached without some practical experience which can best be obtained through an organised society. As your interest is centred in medical missions you should pay a visit to the Bermondsey Medical Mission for Women and Children, 44 Grange Road, Bermondsey, and make yourself acquainted with the work carried on by this excellent institution. In the heart of the densely populated and poverty-stricken district of Bermondsey the Mission has for the past ten years ministered to the sick women and children of the community, and many thousands are now annually dealt with in its dispensary and visited in their own homes. The resident and visiting staff of medical women is assisted by a trained nurse, and by a band of ladies who enter for several months' training before taking up work in other fields. Here you will find an opportunity not only of rendering personal service, but of assisting the Mission by contributing gifts of clothing and many other requisites which are so urgently needed by those seeking relief, or by subscribing to the fund which is being raised for the purchase of an adjacent house and grounds required for future extensions.—"INTERESTED."

### *ACKNOWLEDGMENTS.*

**"Sirod."**—We are much indebted to you for your letter, and for the very useful information contained in it respecting the medical institutions and conditions of nursing at Pau.

**Wayne.**—We thank you for your letter; we are glad to learn that you are about to be received into a suitable Home.

**H. M. B.**—If you will refer to our issue of July 12 you will find the information you require respecting floor polish.

*The Editor is indebted for replies to inquiries from: Dr. C. Donkin, Miss J. Smith, Mr. S. M. Wood, Mr. C. W. Hunt, and Mr. H. P. Cleaver.*

*Communications have been received and attended to from: Miss Stella Hemsley and Sister Herbert.*

### *MISCELLANEOUS.*

**Bed Trolley.**

You can obtain a simple and effective wheeling lifter or trolley from Messrs. J. and J. Taunton, of Sherbourne Road, Birmingham. It is mounted on a pair of castors, and designed for lifting the foot end of the bedstead, to which it is attached by hooks passed under the footbow and over the crossbar below the seat. The action of bringing the lifter into its upright position, in the course of attaching it to the bedstead, lifts the foot end from the floor, and the bed can then be easily wheeled about from place to place. The price of this trolley is 22s. 6d. If you will refer to our issue of May 31 you will find a description and illustration of a similar appliance in use at an American hospital; in this instance, however, two trolleys are used, one at the head and one at the foot of the bed, so that the bed is raised entirely from the floor, and the castors on the back legs of the bed are not brought into use as is the case with the Taunton trolley. These trolleys are mounted on fair-sized wheels instead of castors, which is an advantage, especially if the bed is to be wheeled into the grounds.—"HOSPITAL SECRETARY."

**Charge for Sanatorium Patients.**

WE would advise an arrangement with the local Insurance Committee for patients who are receiving sanatorium benefit at your hospital on the basis of an inclusive *pro rata* charge covering the total cost entailed upon the hospital by the reception of each case. In some institutions the rate agreed has been 27s. 6d. to 30s. a week, but a fixed rate has the objection of not enforcing strict and accurate account keeping which will demonstrably prove the actual cost, and so give a guarantee of careful management throughout.—A. S.

**Sterilising Boxes.**

STERILISING boxes divided into compartments for the purpose of separating sets of dressings have been placed on the market, but they are very rarely used. You can achieve your object by obtaining a number of small boxes, but by far the best and most economical plan is to use a box made to suit the size of your steriliser, and to separate your dressings by packing each set in cloths, labelled in accordance with the character of the dressings enclosed, before placing them in the box; it is then a simple matter, after sterilisation, to remove the set required without endangering the sterility of the whole. A very good type of sterilising box is that which is fitted with a shutter or inlet for steam at top and bottom, which can be packed with cotton-wool; it cannot very well get out of order, and for this reason is more satisfactory than those fitted with a revolving shutter at the circumference.—"NURSE-MATRON."

### Editor's Notices.

**Contributions:**

**Contributions should be written, or preferably typed, on one side of the paper only, and all articles sent in are accepted upon the distinct understanding that they are forwarded to The Hospital only.**

**The Editor cannot undertake to return MSS. not used, but when a stamped directed envelope is enclosed the MSS. may be returned if a special request is made.**

**Accepted articles and paragraphs of news will be paid for after publication at the scale rate.**

**Address:**

To prevent delay all contributions and letters on editorial business must be addressed exclusively to the Editor, "The Hospital," 29 Southampton Street, Strand, London, W.C. It is important that this regulation shall be strictly observed.

**Correspondence:**

Correspondence on all subjects is invited. The name and address of correspondents must be given as a guarantee of good faith, but not necessarily for publication.

**Special Articles:**

Special articles are invited, and questions, inquiries, and paragraphs upon all matters relating to the work, administration and management of general, special, mental, fever, cottage and convalescent hospitals, sanatoria, homes, institutions, societies and organisations for the treatment or care of the sick, injured and dependants of all classes. Every matter affecting the interests and welfare of the staff of all grades working in these institutions will receive special consideration.

**Administrative Medicine, Research and Items of News:**

**Special terms will be paid for approved articles on subjects in this wide field by experts who have made some section of it their special study and interest. We wish to give prominence to every movement tending to advance accuracy of diagnosis, efficiency in treatment, and the development of modern methods to eradicate disease and promote the welfare of the suffering.**

**A Bureau of Information:**

**We invite inquiries and applications for information and help in providing for that numerous class of sufferers who require special care or house-room which they cannot obtain in their own homes or secure for themselves. We cannot, however, prescribe, or recommend practitioners**

### HANDBOOKS
### For TRAINED WORKERS

POCKET-GUIDE SP SERIES

1/- net. 1/1 Post free.

**Bound in Limp Cloth.**
**Suitable Size for the Pocket.**

The works which comprise the S.P. Pocket Guide Series are intended for ready reference, consequently the aim of the Publishers has been to make them as concise and lucid as possible. Each book is written by an expert on the subject of which it treats, and the utmost reliance therefore can be placed in the information it imparts.

**Essentials of Fever Nursing.** By LYTTON MAITLAND, M.D. (Lond.), M.B., B.S., D.P.H. (Camb.).

**Manual and Atlas of Swedish Exercises.** *(With over 60 Illustrations.)* By THOMAS D. LUKE, M.D., F.R.C.S.

**Bandaging Made Easy.** *(Illustrated with over 90 Diagrams.)* By M. HOSKING, Sister-in-Charge, Tredegar House, Bow, E.

**How to Write and Read Prescriptions.** By LYTTON MAITLAND, M.D. (Lond.), M.B., B.S., D.P.H. (Camb.).

**Principal Drugs and their Uses.** By A PHARMACIST.

**Asepsis and How to Secure It.** By H. W. CARSON, F.R.C.S.

**Treatment after Operations.** *Rules for Nursing after General and Special Operations.* By MARY WILES.

**The Nurse's Duties before and during Operations.** By MARGARET FOX, Matron, Prince of Wales's General Hospital, Tottenham.

**Other works, in amplification of this Series, will be announced in due course.**

*Published by*

**The SCIENTIFIC PRESS, Ltd.**

**28/29 Southampton Street, STRAND, LONDON, W.C.**

**400 NEW WORDS**

have been added to the

**NEW EDITION**

of

### The Nurse's Pronouncing Dictionary

**OF MEDICAL TERMS AND NURSING TREATMENT**

Compiled by **HONNOR MORTEN.**

Eighth Edition, thoroughly Revised and much Enlarged.

Edited by **MARY I. BURDETT.**

The new issue of this well-known Dictionary is the **most complete work** of the kind published. It has been completely revised and some hundreds of new words have been added, consequently the utmost reliance can be placed in the information it contains.

To be up-to-date you should GET A COPY TO-DAY.

**Price, in cloth, 2/- post free; in leather, 2/6 net, by post 2/8.**

*Of all Booksellers, or of—*

**The Scientific Press, Ld.**
*(The Nurses' Publishers),*
**28/29 Southampton Street, STRAND, LONDON, W.C.**

By Post **5/4**

**It is possible to secure "the most up-to-date and Complete Guide to Nursing that has yet been written."**

*Vide THE NURSING MIRROR.*

### A HANDBOOK FOR NURSES

**With Examination Questions based upon the contents of the Chapters.**

**By J. K. WATSON, M.D.Edin., C.M.**

**5th Edition, Entirely Revised, and Enlarged to 544 pages**

The title of this book very inadequately describes its scope. It would be more accurately entitled "*THE* Handbook for Nurses," since the information it contains renders it of real and genuine service to the modern Nurse. The fact that **31,500** copies of former editions have been sold is indisputable evidence of its value. The work forms practically an Encyclopædia of Medical, Surgical and Monthly Nursing, and there are few, if any, Nursing details that do not find representation in its pages. If you have not seen the New Edition we would strongly advise you to take the earliest opportunity of doing so.

It is obtainable of all Booksellers, or direct from . . . .

**THE NURSES' PUBLISHERS,**

**28/29 SOUTHAMPTON STREET, STRAND - LONDON, W.C.**

